# Silicon Improves Chilling Tolerance During Early Growth of Maize by Effects on Micronutrient Homeostasis and Hormonal Balances

**DOI:** 10.3389/fpls.2018.00420

**Published:** 2018-04-26

**Authors:** Narges Moradtalab, Markus Weinmann, Frank Walker, Birgit Höglinger, Uwe Ludewig, Guenter Neumann

**Affiliations:** ^1^Institute of Crop Science (340h), University of Hohenheim, Stuttgart, Germany; ^2^Institute of Phytomedicine (360), University of Hohenheim, Stuttgart, Germany

**Keywords:** silicon, micronutrients, germination, chilling stress, maize, oxidative stress

## Abstract

Low soil temperature in spring is a major constraint for the cultivation of tropical and subtropical crops in temperate climates, associated with inhibition of root growth and activity, affecting early growth and frequently plant performance and final yield. This study was initiated to investigate the physiological base of cold-protective effects induced by supplementation with silicon (Si), widely recommended as a stress-protective mineral nutrient. Maize was used as a cold-sensitive model plant, exposed to chilling stress and low root-zone temperature (RZT) during early growth in a lab to field approach. In a pot experiment, 2–weeks exposure of maize seedlings to low RZT of 12–14°C, induced leaf chlorosis and necrosis, inhibition of shoot and root growth and micronutrient limitation (particularly Zn and Mn). These phenotypes were mitigated by seed treatments with the respective micronutrients, but surprisingly, also by Si application. Both, silicon and micronutrient treatments were associated with increased activity of superoxide dismutase in shoot and roots (as a key enzyme for detoxification of reactive oxygen species, depending on Zn and Mn as cofactors), increased tissue concentrations of phenolics, proline, and antioxidants, but reduced levels of H_2_O_2_. These findings suggest that mitigation of oxidative stress is a major effect of Zn, Mn, and Si applied as cold stress protectants. In a soil–free culture system without external nutrient supply, Si significantly reduced large leaching losses of Zn and Mn from germinating seeds exposed to low-temperature stress. Silicon also increased the translocation of micronutrient seed reserves to the growing seedling, especially the Zn shoot translocation. In later stages of seedling development (10 days after sowing), cold stress reduced the root and shoot contents of important hormonal growth regulators (indole acetic acid, gibberellic acid, zeatin). Silicon restored the hormonal balances to a level comparable with non-stressed plants and stimulated the production of hormones involved in stress adaptation (abscisic, salicylic, and jasmonic acids). Beneficial effects of Si seed treatments on seedling establishment and the nutritional status of Zn and Mn were also measured for a field-grown silage maize, exposed to chilling stress by early sowing. This translated into increased final biomass yield.

## Introduction

In the context of global warming, there is an increasing trend for the cultivation of crops with tropical and subtropical origins, such as maize, soybean, *Miscanthus* or *Sorghum* also in temperate climates, e.g., in Central Europe. Under these conditions, early sowing is required for efficient use of the comparatively shorter vegetation periods and to escape from detrimental effects of summer drought (Hund et al., 2004). However, low temperature and cold and wet soils during early spring represent major constraints for the cultivation of tropical crops bearing the risk of poor germination, impaired seedling establishment, and reduced nutrient acquisition due to limited root growth and activity. This is frequently associated with poor vegetative plant development, reduced stress resistance and finally reduced crop yield (Duncan and Hesketh, 1968; Muldoon et al., 1984; Imran et al., 2013), although under favorable conditions short cold periods can be tolerated and later compensated until final harvest (Saeidnejad et al., 2012). In plant species, such as maize, with optimum temperatures of 25–30°C for germination and plant growth, even moderately low soil temperatures <15°C are already detrimental (Cutforth et al., 1986; Kasper and Bland, 1992).

Due to root growth limitation and slow diffusion rates, plant availability, and acquisition of sparingly soluble nutrients, such as P, ${\text{NH}}_{4}^{+}$, K, Fe, Zn, Mn, and Cu, is particularly affected by low soil temperatures (Duncan and Hesketh, 1968; Kramer and Boyer, 1995; Wan et al., 2001). Therefore, placement of these nutrients close to the seedling roots or as seed treatments are among the most widespread practical measures to counteract detrimental effects of low soil temperatures on seedling establishment and early growth of maize (Imran et al., 2013; Bradáčová et al., 2016; Nkebiwe et al., 2016). Meanwhile, starter applications of ammonium phosphates by shallow subsurface placement below the seeds (Nkebiwe et al., 2016) belong to the standard fertilization strategies employed for maize production systems in temperate climates. Micronutrients with stress-protective functions, such as Zn and Mn, are frequently applied as foliar sprays. However, this is not possible in the seedling stage and usually, the formulations are more expensive than soil fertilizers. More cost-effective placement strategies including seed dressings are increasingly employed to promote stress resistance, early growth, and crop establishment (Farooq et al., 2012; Bradáčová et al., 2016). Similar stress-protective effects have been recorded also for amendments with Si (Liang et al., 2015).

When plants are exposed to environmental stress factors, such as chilling, drought or salinity, an imbalance between production and detoxification of reactive oxygen species (ROS) promotes accumulation of ROS, which induces oxidative damage to cellular components (Gong et al., 2005). Many studies have highlighted a role of Si in the suppression of oxidative damage in various plant species under a wide range of stress conditions. Protective effects have been reported against drought stress in wheat (Gong et al., 2005, 2008; Pei et al., 2010), salinity and boron toxicity in tomato, spinach (Al-aghabary et al., 2005; Gunes et al., 2007), cotton (Gossett et al., 1994), and barley (Liang et al., 2003; Inal et al., 2009), or low temperature stress in wheat (Liang et al., 2008) and cucumber (Liu et al., 2009). Mitigation of oxidative stress by Si treatments has been related to increased expression of enzymatic ROS detoxification systems, such as superoxide dismutases, catalases, peroxidases, ascorbate peroxidase, and increased accumulation of antioxidants (phenolics, proline, ascorbic acid), similar to the effects mediated by stress-protective micronutrients, such as Zn, Mn, Fe, and Cu (Cakmak, 2000; Datnoff et al., 2007). However, the links between Si application and induction of the protective mechanisms against oxidative stress are still largely unknown.

Therefore, this study was designed to investigate more in detail the physiological background of Si effects on cold stress mitigation during early growth of maize. We hypothesized that the protective role of silicon is related to the homeostasis of cold stress-protective micronutrients, such as Zn and Mn (Imran et al., 2013; Bradáčová et al., 2016). In a comparative investigation, Zn, Mn, and Si were applied as seed treatments or by starter fertigation to maize plants, subsequently exposed to low temperatures of 12–14°C on a silty loam soil taken from a field site of maize cultivation and also in a soil-free culture system. Plant growth, symptoms of oxidative leaf damage and the mineral nutritional status in different plant organs were documented in relation with the expression of various physiological stress indicators (production of H_2_O_2_, activities of superoxide dismutase and peroxidase, accumulation of antioxidants, phenolics, and proline) and with changes in hormonal balances. Finally, a preliminary field experiment was conducted on the same soil, where low-temperature stress was provoked due to early sowing at the mid of April. This allowed evaluating the expression of micronutrient and Si effects and their impact on final yield under practical conditions.

## Materials and methods

### Plant cultivation

#### Soil culture experiment

*Zea mays* L cv. Colisee was used as test plant. Soil material (silty-loam, pH 6.9) was derived from the Ap horizon of a maize cultivation field site at the Hohenheim University experimental station Ihinger Hof, Renningen, Germany (Supplementary Table 1). After sieving with 2 mm mesh size, fertilization was performed with Ca(NO_3_)_2_, 100 mg N kg^−1^ DM; Ca(H_2_PO_4_)_2_, 80 mg P kg^−1^ DM; K_2_SO_4_, 150 mg K kg^−1^DM and MgSO_4_, 50 mg Mg kg^−1^ DM. For improvement of the soil structure, the fertilized soil was mixed with quartz sand (ratio 2:1). Plastic pots were filled with 1,800 ml of this soil substrate and inserted into a cooling system, designed to control the root zone temperature of plants. An immersion water bath circulator (Thermomix 1480/Frigomix 1497, Braun, Melsungen, Germany) was connected to the cooling system, equipped with a closed pipe system which was installed into moist peat substrate to circulate the refrigerating fluid through the moist peat layer surrounding the culture vessels (Bradáčová et al., 2016). The plants were regularly watered to 70% of substrate water holding capacity (WHC) with deionized water and cultivated for 2 weeks at a root zone temperature of 20–22°C, 2 weeks at low root zone temperature (12–14°C), followed by a 2 weeks' recovery phase at 20–22°C.

Silicon was supplied as silicic acid (H_4_SiO_4_) prepared by passing K_2_SiO_3_ through a column filled with a cation–exchange resin (Amberlite IR−120, H^+^ form, Sigma Aldrich, Germany) according to Maksimovic et al. (2007) and Pavlovic et al. (2013). Silicon fertigation was performed at a dosage of 40 mg H_4_SiO_4_ kg^−1^ soil DM applied with a pipette close to the plant, directly on top of the soil substrate in four weekly intervals, starting at the sowing date. Seed dressing with Zn and Mn was performed with commercial formulations: Lebosol® Mn500 SC and Lebosol® Zn700 SC (Lebosol® Dünger GmbH, Ermstein, Germany) according to the manufacturer instructions. Lebosol® Zn700 SC: 2 ml 4,000 seeds^−1^, Lebosol® Mn500 SC: 4 ml 4,000 seeds^−1^.

#### Soil–free culture experiment

Germination of maize seeds (*Z. mays* cv. Colisee), surface–sterilized by 1 min soaking in ethanol (99% v/v), was performed in pre-sterilized Petri dishes (9 cm diameter) on moist filter paper (10 seeds per petri dish). The seeds were soaked with 3 mL deionized water (–Si control) or 3 mL freshly prepared H_4_SiO_4_ [1.0 mM Si] (Maksimovic et al., 2007; Pavlovic et al., 2013), respectively with four replicates per treatment. The optimum level of Si seed application was determined in a pilot experiment with different levels of Si supply (0–3.0 mM Si, Supplementary Figure 1). Covered Petri dishes were placed into a laboratory incubator (AtmoCONTROL, ICP, 750 Memmert GmbH, Schwabach, Germany) for 3 days in the dark at 18°C in thin unsealed plastic bags to minimize evaporation. For further seedling development, the germinated seeds were placed on the upper edge of rolled filter papers (10^*^60 cm; 2 seeds per roll), moistened with 100 ml distilled water or H_4_SiO_4_ solution [1 mM Si] at 3 and 5 days after sowing (DAS). The filter rolls were transferred into the laboratory incubator and incubated for 7 days at 12°C (16/8 h light/dark period and relative humidity of 60%, with a light intensity of 200 μmol·m^−2^·s^−1^ or at 18°C outside the incubator.

#### Field experiment

In 2016, a field experiment was established for silo maize production (*Z. mays* cv. *Rolandinio*) at the experimental station “Ihinger Hof” University of Hohenheim, Renningen, Germany (soil properties are specified in Supplementary Table 1). To increase the probability of low-temperature stress in spring, sowing was conducted on April 22nd. Accordingly, a mean air temperature of 13.6°C during April and May was recorded associated with heavy rainfall (469 mm) leading to cold and wet soil conditions during germination and emergence. Fertilization was conducted prior to sowing by broadcast application with subsequent soil incorporation of stabilized ammonium sulfate NovaTec® solub 21: 161 kg N ha^−1^ (Compo–Expert, Münster, Germany) and by underfoot placement of di-ammonium phosphate (29 kg N, 32 kg P ha^−1^). Seed treatments were performed as seed dressings with Zn and Mn (Lebosol ® Mn500 SC and Lebosol® Zn700 SC (Lebosol® Dünger GmbH, Ermstein, Germany) as described in experiment (1), and as seed priming with 1 mM potassium silicate (KSi, PottaSol, BioFa, Münsingen Germany) with a 24 h seed soaking period and re-drying during 24 h at 28 ± 2°C. A seed water priming treatment without Si was included as a control. Sowing was performed on April 22nd with a sowing density of 9 seeds m^−2^, a row distance of 75 cm and a sowing depth of 6–7 cm. Foliar Si application (Vitanica® Si, Compo, Münster, Germany 16 L ha^−1^ + 100 ml ha^−1^ Greemax® Stallen Bio Agro AG, Basel, Switzerland) was conducted with a backpack sprayer (Solo®, Sindelfingen–Maichingen, Germany) at 69, and 75 DAS. Due to the extremely cold and wet soil conditions by the end of April, seedling emergence was severely biased, particularly in the untreated control variants. Therefore, after recording of emergence rates at 41 days after sowing (DAS), re–sowing was performed in the heavily affected plots to maintain a comparable level of inter-plant competition for light, water, and nutrients within the rows during the rest of the culture period. Final harvest was conducted at 214 DAS.

### Plant analysis

Visual scorings of leaf chlorosis, necrosis, and anthocyanin formation and determination of shoot height was performed for all experiments. Reflectometric leaf chlorophyll measurements were performed with a SPAD meter (Konica Minolta INC, Osaka, Japan). Estimates of damaged leaf area were obtained by the equation, leaf area (cm^2^) = x/y, where x is the weight (g) of the area covered by leaf drawings on a transparent millimeter graph paper, and y is the weight of 1 cm^2^ of the same graph paper (Pandey and Singh, 2011). Based on these data, the percentage of the necrotic area was calculated. After final harvest, root and shoot dry matter was determined after 60°C oven-drying. Root length measurements were performed by digital image analysis using the WinRHIZO root analysis software package (Regent Instruments Inc., Quebec, Canada).

### Analysis of mineral nutrients

One hundred milligrams of dried, milled shoot material were ashed for 5 h in a muffle furnace at 500°C. After cooling, the samples were digested twice with 1 mL of 3.4 M HNO_3_ and evaporated until dryness to precipitate SiO2. The ash was dissolved in 1 mL of 4 M HCl, subsequently diluted 10 times with hot deionized water, and boiled for 2 min to convert meta-, and pyro-phosphates to orthophosphate. After addition of 0.1 mL Cs/La buffer to 4.9 mL ash solution, Mg, Fe, Mn, and Zn concentrations were measured by atomic absorption spectrometry (ATI Unicam Solaar 939, Thermo Electron, Waltham, USA). Spectrophotometrical determination (Hitachi U−3300 spectrophotometer, Hitachi LtD. Corporation Japan) of orthophosphate was conducted after addition of molybdate-vanadate color reagent according to the method of Gericke and Kurmis (1952). K and Ca were measured by flame emission photometry (ELEX 6361, Eppendorf, Hamburg, Germany). Silicon was analyzed by ICP–OES (Vista–PRO, Varian Inc., Palo Alto, USA). For the digestion, 0.250 g of sample DM was suspended in 1 mL of H_2_O and 2.5 mL of conc. HNO_3_. The digestion is carried out by means of microwave–heated pressure digestion with HNO_3_ and HF at 220°C in a digestion system Ultra clave II (MLS GmbH, Leutkirch, Germany). The digestion took place over 20 min, the entire digestion program with heating and cooling phases comprised 2 h. After digestion, 0.5 ml HF solution (1% v/v) was added to dissolve sparingly soluble silicates. The solutions were adjusted to 10 mL with distilled H_2_O (VDLUFA Method book VII, 2011) and used for ICP–OES analysis.

### Superoxide dismutase assay

The superoxide dismutase (SOD, EC 1.15.1.1) assay was optimized for root and shoot tissues of maize according to the method described by Beauchamp and Fridovich (1971) and modifications suggested by Giannopolitis and Ries (1977) and Hajiboland and Hasani (2007). One hundred milligrams of fresh plant material, frozen in liquid nitrogen and stored at −80°C, were ground with a pre-cooled mortar and pestle, and homogenized in 1.5 ml extraction buffer containing 25 mM HEPES pH 7.8 and 0.1 mM EDTA. After centrifugation at 10,000 × g (4°C for 10 min), aliquots of supernatant were transferred into 2 ml reaction tubes and kept on ice. For preparation of the reaction mixture, 1 ml cuvettes covered with aluminum foil for light protection, were filled with 300 μl 62.5 mM HEPES, 75 μl 1.0 mM EDTA, 75 μl 120 mM Na_2_CO_3_, 75 μl 120 mM L-methionine, 150 μl 750 μM nitro-blue tetrazolium (NBT), and 100 μl of plant extract. Finally, 225 μl of 10 μM riboflavin was added. The light reaction was started by removing the aluminum foil, exposing the samples to a light source (8000 Lux) for 25 min. During the light phase, NBT is reduced to a dark blue formazan, measured spectrophotometrically (Spectrophotometer U−3300, Hitachi, Tokyo, Japan) at a wavelength of 650 nm. The final SOD activity, which inhibits the NBT reduction, was calculated as the difference between the absorbance of the sample and a control without plant extract, divided by 50% absorbance of the control. The specific SOD activity was expressed as SOD units per mg total protein. Total, protein content was determined according to Bradford (1976).

### Peroxidase assay

Peroxidase (POD, EC1.11.1.7) activity was determined using the guaiacol test (Chance and Maehly, 1955; Hajiboland and Hasani, 2007). The tetra-guaiacol formed during the reaction is measured photometrically at 470 nm. The enzyme was extracted from fresh leaf material (100 mg) by 10 mM phosphate buffer (pH 7.0) and centrifuged 1,000 g for 10 min. The test mixture (1 mL) contained 10 mM phosphate buffer (300 μL, pH 7.0), 5 mM H_2_O_2_ (300 μL), and 4 mM guaiacol (300 μL). The reaction was started by addition of the enzyme extract (100 μL) at 25°C. The formation of tetraguaiacol was recorded over a reaction period of 5 min and the specific enzyme activity was expressed in μmoles tetraguiacol formation mg^−1^ total protein.

### Determination of H_2_O_2_

Hydrogen peroxide levels were determined as described by Harinasut et al. (2003). Leaf tissues (100 mg fresh weight) were homogenized in an ice bath with 5 ml 0.1% (w/v) trichloroacetic acid. The homogenate was centrifuged at 12,000 g for 15 min. 0.5 ml of the supernatant was added to 0.5 ml of 10 mM potassium phosphate buffer (pH 7.0) and 1 ml of 1 M KI. The absorbance of the supernatant was recorded at 390 nm. The concentration of hydrogen peroxide was determined using a standard curve ranging from 0 to 120 μM of H_2_O_2_.

### Determination of total soluble sugars, phenolics, and proline

For determination of soluble sugars, leaf and root samples were homogenized in 100 mM phosphate buffer (pH 7.5) at 4°C. After centrifugation at 12,000 g for 15 min, the supernatant was used for determination of total soluble sugars (Yemm and Willis, 1954). An aliquot of the supernatant was mixed with anthrone-sulfuric acid reagent (Yemm and Willis, 1954) and incubated for 10 min at 100°C. After cooling, the absorbance was recorded at 625 nm. A calibration curve was created using glucose as external standard (Merck, Darmstadt, Germany). Total phenolics concentration was determined spectrophotometrically at 750 nm, using the Folin method (Hajiboland et al., 2017). For determination of proline, samples were homogenized with 3 % (v/v) sulfosalicylic acid and the homogenate was centrifuged at 3,000 g for 20 min. The supernatant was treated with acetic acid and acid ninhydrin, boiled for 1 h, and then the absorbance was determined at 520 nm. Proline (Sigma-Aldrich, Munich, Germany) was used for the production of a standard curve (Bates et al., 1973).

### Determination of total soluble anthocyanins and flavonoids

Determination of anthocyanins was conducted spectrophotometrically at 510 nm according to Plessi et al. (2007). One hundred milligrams of fresh shoot material was extracted with 2 ml methanol/HCl conc. (98:2 v/v). After centrifugation at 12,000 × g for 10 min, each 0.5 mL of the supernatant was used for spectrophotometric determination by using a pH differential method at pH 1 and pH 4.5 adjusted with 4.5 ml of MES buffer After 5 h incubation at 4°C the absorbance was read at 510 nm from each group. The results were calculated as cyanidine-3-glycoside equivalents, using the formula ΔA^*^MW^*^DF^*^V^*^100/ε^*^Wt, where ΔA is Abs (pH 1) – Abs (pH 4.5), MW = molecular weight of cyanidine-3-glycoside (484.83 g mol^−1^), DF = dilution factor, V = final volume of the supernatant (0.5 ml), ε = molar absorbance factor of cyanidine-3-glycoside (26,900 Mm^−1^ cm^−1^), L = diameter of the light path [cm], and Wt = sample fresh weight (0.1 g). Total leaf flavonoids were determined in methanolic extracts according to Hajiboland et al. (2014). One hundred milligrams of fresh leaf material was extracted in AlCl_3_-methanol (2%, w/v) and after centrifugation at 12,000 × g for 10 min, the supernatant was used for determination at 415 nm with quercetin (Sigma, Munich, Germany) as an external standard.

### Determination of total antioxidants

The 1,1-diphenyl-2-picrylhydrazyl radical (DPPH •) has been used to evaluate the free radical scavenging activity of antioxidants (Panico et al., 2009). The DPPH solution was prepared by adding 2.37 mg DPPH (Sigma-Aldrich, Munich, Germany) in 2 ml 99% ethanol. One hundred milligrams of fresh leaf samples were grinded in 1 ml of extraction solution (1:1 Ethanol:Water). After centrifugation at 12,000 g for 10 min at 4°C, 50 μl supernatant was used in a reaction cell which contained 50 μl freshly prepared 3 mM DPPH solution and 900 μl ethanol (99%). After incubation for 10 min in a dark room at 25°C, the absorbance was determined at 515 nm. A reference solution contained 50 μl DPPH solution and 950 μl of ethanol (99%). The decline in absorbance at 515 nm was recorded for each sample and the quenching percentage of the DPPH radical was calculated based on the observed decrease in absorbance using the formula:

% Inhibition = [(A_0_-A_1_)/A_0_] × 100, where A_0_ is the absorbance value of the DPPH • blank solution and A_1_ is the absorbance value of the sample solution.

### Determination of phytohormones by UHPLC-MS analysis

Frozen maize tissue samples (shoot, roots) of 1 g of were ground to a fine powder with liquid nitrogen and extracted twice with 2.5 ml of 80% methanol in falcon tubes. Thereafter, the samples were further homogenized by ultrasonication (Micra D-9 homogenizer, Art, Müllheim Germany) for 1 min and 15 s at 10,000 rpm. Two milliliters of the methanol extracts were transferred to microtubes and centrifuged at 5,645 × g for 5 min. Thereafter, 350 μl of the supernatant was mixed with 700 μl ultra-pure water and centrifuged at 5,645 × g for 5 min. The supernatant was cleaned by membrane filtration (Chromafil® O-20/15 MS) and transferred to HPLC vials. UHPLC-MS analysis was carried out on a Velos LTQ System (Thermo Fisher Scientific, Waltham, Massachusetts, USA) fitted with a Synergi Polar column, 4 μ, 150 ^*^ 3.0 mm, (Phenomenex, Torrance, California, USA). The injection volume was 3 μL and the flow rate was adjusted to 0.5 ml min^−1^ for gradient elution with mobile phase (A): water and 5% acetonitrile; mobile phase (B): acetonitrile and a gradient profile of: 0–1 min, 95% A, 5% B, 11–13 min, 10% A, 90% B, 13.1 min, 95% A, 5% B, 16 min 95% A, 5% B). All standards were purchased from Sigma Aldrich, (Sigma Aldrich, St. Louis, Missouri, USA) including (+/–)-jasmonic acid; 3-indoleacetic-acid, gibberellic acid, (+/–)-abscisic acid; trans-zeatin; salicylic acid.

### Statistical analyses

The study was carried out in a completely randomized design for pot experiments and a randomized block design for the field experiment. Data are presented as means ± SE. For statistical analysis of significant differences between treatment groups, a one-way ANOVA followed by a Tukey–test (*p* < 0.05 significance level) were performed using the Sigma-Plot software 10.0 (Systat Software GmbH, Erkrath, Germany). For the statical evaluation of yield data from the field experiment, t-grouping instead of the Tukey test was applied as recommended by Mudra (1958).

## Results

### Soil culture experiment

**Figure 1:**
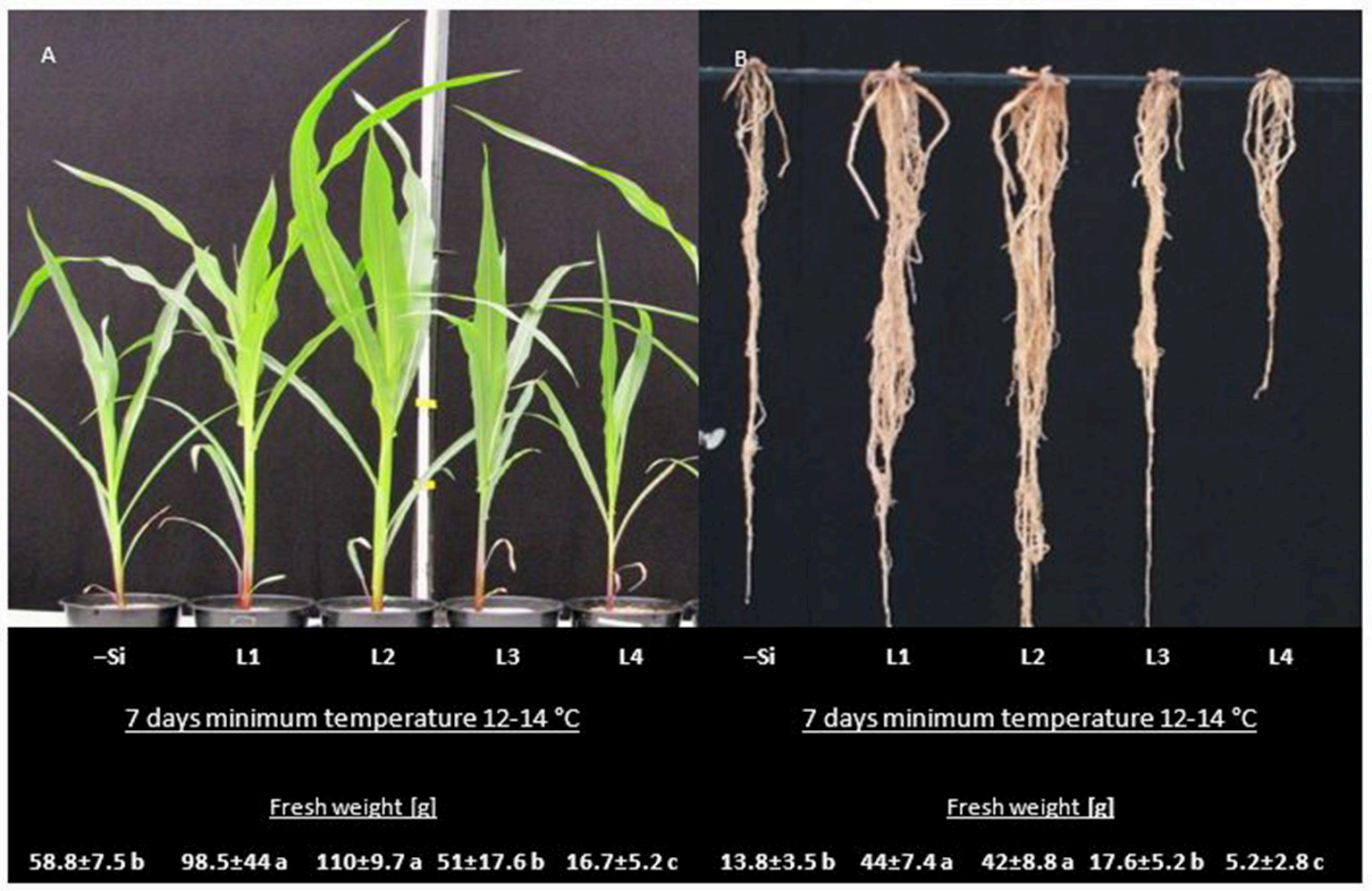
**(A)** Shoot and **(B)** root development and biomass of maize plants including untreated control (–Si), and different levels (L) of Si (silicic acid) fertigation (L1: 25, L2: 85, L3:1,000 and L4: 10,000 mg Kg^−1^ soil DM). Culture period: 8 weeks under greenhouse conditions with 7 days minimum temperature of 12–14°C. Biomass data represent mean values ± SE of four replicates. Significant differences (*P* < 0.05) are indicated by different letters.

In the first experiment, soil-grown maize seedlings were exposed to 14 days reduced root zone temperature of 12–14°C in a root cooling system. Cooling of the roots started at 2 weeks after sowing, followed by a 14-d recovery period. Seed treatments were performed with a commercial Zn/Mn seed dressing formulation. Silicon was applied as silicic acid (40 mg kg^−1^ dry soil) by fertigation in four weekly intervals. The optimal dosage for silicic acid application has been determined in a pilot experiment (Figure 1).

#### Plant growth and development

**Figure 2:**
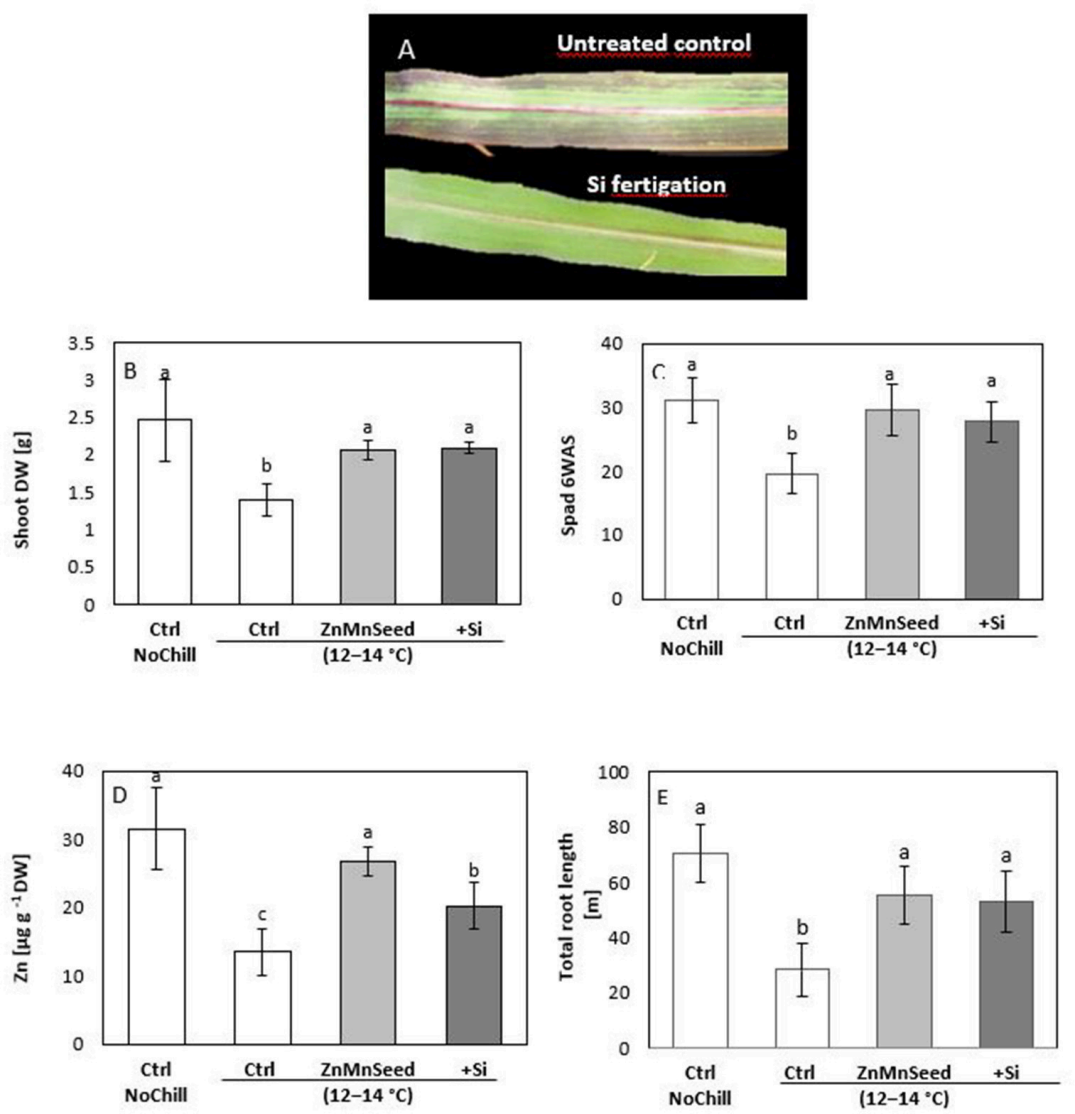
**(A)** Leaf chlorosis, necrosis, and anthocyanin formation, **(B)** shoot dry weight (DW), **(C)** SPAD values, **(D)** shoot Zn concentration and **(E)** total root length of maize plants exposed to a 2-weeks period of reduced root zone temperature (RZT, 12–14°C) on a silty loam soil, pH 6.9. Un-cooled control: (Ctrl NoCill) and low RZT variants including untreated control (Ctrl); Zn Mn seed dressing (ZnMnSeed), and silicon (H_4_SiO_4_) fertigation (+Si). Means of three replicates. Significant differences (*P* < 0.05) are indicated by different characters.

**Figure 3:**
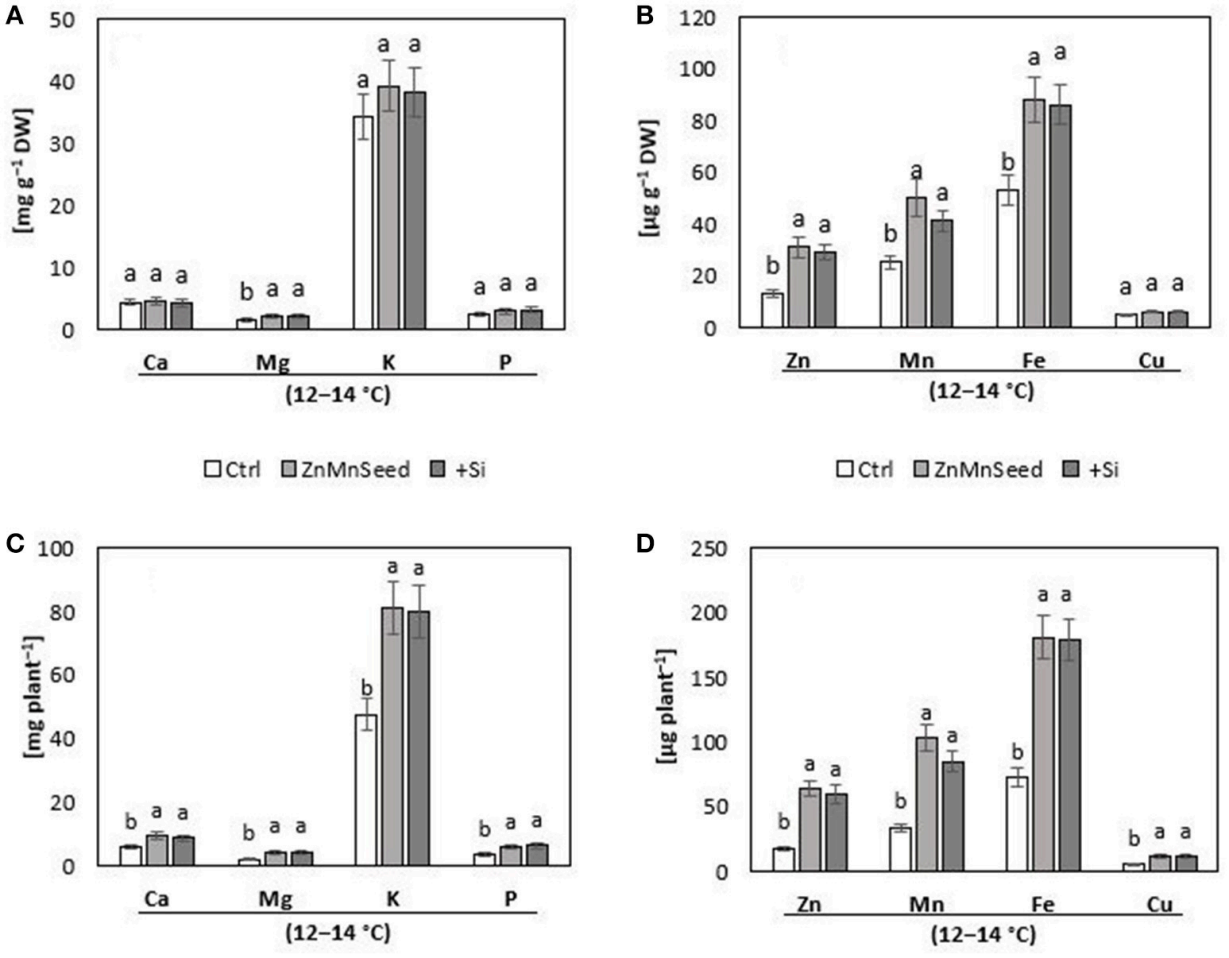
**(A,B)** Concentrations and **(C,D)** contents of macro-, and micro-nutrients in shoots of maize plants (cv Colisee) exposed to a 2-weeks period at reduced root zone temperature (12–14°C) on a silty loam soil, pH 6.9. Untreated control: (Ctrl); Zn Mn seed dressing: (ZnMnseed), and silicon (H_4_SiO_4_) fertigation (Si). Means of three replicates. Significant differences (*P* < 0.05) are indicated by different characters.

Confirming the results of our earlier studies (Bradáčová et al., 2016), 2 weeks exposure of maize plants to low RZT of 12–14°C was associated with induction of leaf chlorosis (Figures 2A,C) necrosis, formation of stress anthocyanins (Figure 2A) limited shoot and root growth (Figures 2B,E) and an impaired micronutrient status (particularly Zn and Mn) below the deficiency thresholds (Figures 2D, 3A; Bergmann, 1988). The stress responses and micronutrient deficiencies were mitigated by seed treatments with the respective micronutrients, but surprisingly, also by Si application (Figure 2). Shoot biomass and chlorophyll contents determined by SPAD measurements were increased by ~50% (Figures 2B,C) and total root length even by 90% (Figure 2E).

#### Mineral nutritional status

Macronutrient analysis of the shoot tissue revealed no significant differences for the P, Ca, and K concentrations (Figure 3A). While the Ca and K status was in the sufficiency range, low concentrations were recorded for P and Mg. Seed dressing with Zn/Mn or Si fertigation significantly increased particularly the Mg status of the plants (Bergmann, 1988). The shoot micronutrient concentrations of the untreated control were 0.013 mg g^−1^ DM for Zn and 0.025 mg g^−1^ DM for Mn (Figure 3B) which is below the reported deficiency thresholds (Bergmann, 1988). The concentrations increased to the sufficiency range in response to the Zn/Mn seed dressing treatment, but interestingly also after Si fertigation (Figure 3A). The Fe and Cu status was low but not critical, without significant differences between the treatments (Figure 3A). Shoot Si was significantly increased only by Si fertigation (Table 1). In contrast to the nutrient concentrations, as indicators for the plant nutritional status, total shoot contents of all investigated nutrients were significantly increased by the treatments with the chilling stress protectants Si and Zn/Mn (Figures 3C,D). This demonstrates that the Si and Zn/Mn applications generally improved nutrient acquisition and any surplus of mineral nutrients was readily transformed into biomass production (Figure 2B).

**Table 1:** Superoxide dismutase activity (SOD), peroxidase activity (POD), and H_2_O_2_ concentrations in root tissue and SOD, total protein, total antioxidant capacity, total soluble phenolics, total soluble flavonoids, proline, anthocyanin, total soluble sugars, and Si concentrations in the shoot tissue of maize plants (cv Colisee) exposed to a 2-weeks period at reduced root zone temperature (12–14°C) on a silty loam soil, pH 6.9.

**Tissue**	**Determination**	**Ctrl**	**ZnMnSeed**	**+Si**
Root	SOD [U mg^−1^ protein]	85.94 ± 8.68b	124.52 ± 12.44a	125.29 ± 12.62a
	POD [μmol tetra guaicol mg^−1^ protein]	54.19 ± 5.51b	99.76 ± 10.07a	92.51 ± 9.34a
	H_2_O_2_ [μmol g^−1^ FW]	85.70 ± 8.66a	39.81 ± 4.07b	32.50 ± 3.34b
Shoot	SOD [U mg^−1^ protein]	10.06 ± 1.10b	26.51 ± 2.74a	28.02 ± 2.89a
	Total protein [mgg^−1^ FW]	6.58 ± 0.75b	8.39 ± 0.93a	8.19 ± 0.91a
	Total antioxidants [%]	69.11 ± 7.00b	91.20 ± 9.21a	88.13 ± 8.90a
	Phenolics [mg gallic acid equivalents g^−1^ FW]	50.31 ± 5.12*b*	79.15 ± 8.01a	78.19 ± 7.91a
	Flavonoids [mg g^−1^ FW]	2.25 ± 0.32b	3.41 ± 0.43a	3.35 ± 0.43a
	Proline [μmol g ^−1^ FW]	0.92 ± 0.18b	1.68 ± 0.26a	1.89 ± 0.28a
	Anthocyanin [μmol cyanidine-3-glucoside equivalents g^−1^ FW]	9.88 ± 1.08a	6.40 ± 0.73b	6.16 ± 0.71*b*
	Sugar [mg g^−1^ FW]	19.23 ± 2.01b	21.18 ± 2.21ab	24.15 ± 2.51a
	Si [mg g^−1^ DW]	2.70 ± 0.36b	2.88 ± 0.38b	4.15 ± 0.51a

*Untreated control: (Ctrl); Zn Mn seed dressing: (ZnMnSeed), and silicon (H_4_SiO_4_) fertigation: (+Si). Means of three replicates. Significant differences (P < 0.05) are indicated by different characters*.

#### Oxidative stress indicators

In the root tissue directly exposed to low-temperature stress, Zn/Mn and Si applications increased the activities of superoxide dismutase (SOD, Table 1) and peroxidase (POD, Table 1) by 45–46 and 71–84%, respectively. These enzymes are involved in oxidative stress defense and accordingly, H_2_O_2_ concentrations declined by 54–63% (Table 1). In the shoot, SOD activities even increased by 179–183% (Table 1). Shoot concentrations of total proteins, antioxidants, total phenolics, total flavonoids, and proline concentrations were also strongly increased (Table 1) by both the Zn/Mn and Si treatments, while leaf anthocyanins, significantly declined (Table 1). In addition, the concentration of total soluble sugars of shoot tissue was significantly enhanced by Si treatment (Table 1).

### Soil–free culture experiment

Although Si induced a recovery of maize seedling growth after exposure to low RZT, which was associated with improved root development and mitigation of Zn and Mn deficiency (Figure 3, Table 1) we hypothesized that Si may exert also effects on improvement of the micronutrient status independent of root uptake. Thus, a second experiment was conducted to separate the root–mediated nutrient acquisition from internal redistribution of Zn and Mn from the seed to the establishing seedling. This was achieved by exposing maize seedlings to low temperature stress (7 d, 12°C) in a soil–free culture system with and without Si application via seed soaking (3 d during germination at 18°C), where the seedlings were exclusively dependent on their nutrient seed reserves and root uptake was excluded.

**Figure 4:**
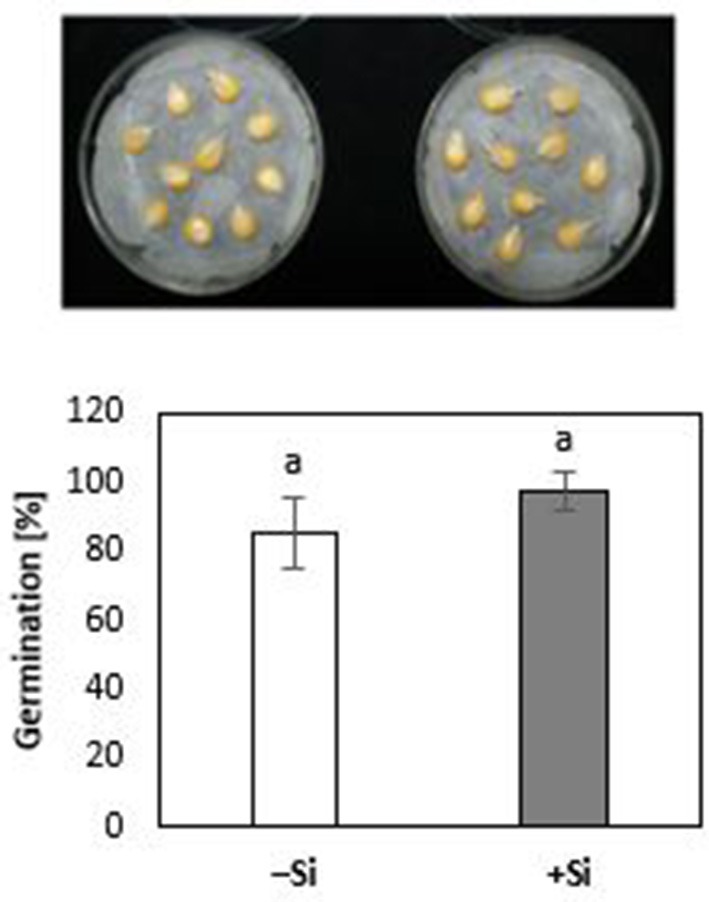
Germination rate [% radicle emergence at 3 DAS; 18°C) of maize seedlings (cv Colisee) on moist filter paper with (+Si) or without (–Si) 3-days seed soaking treatments with 0.1 mM Si (H_4_SiO4).

**Figure 5:**
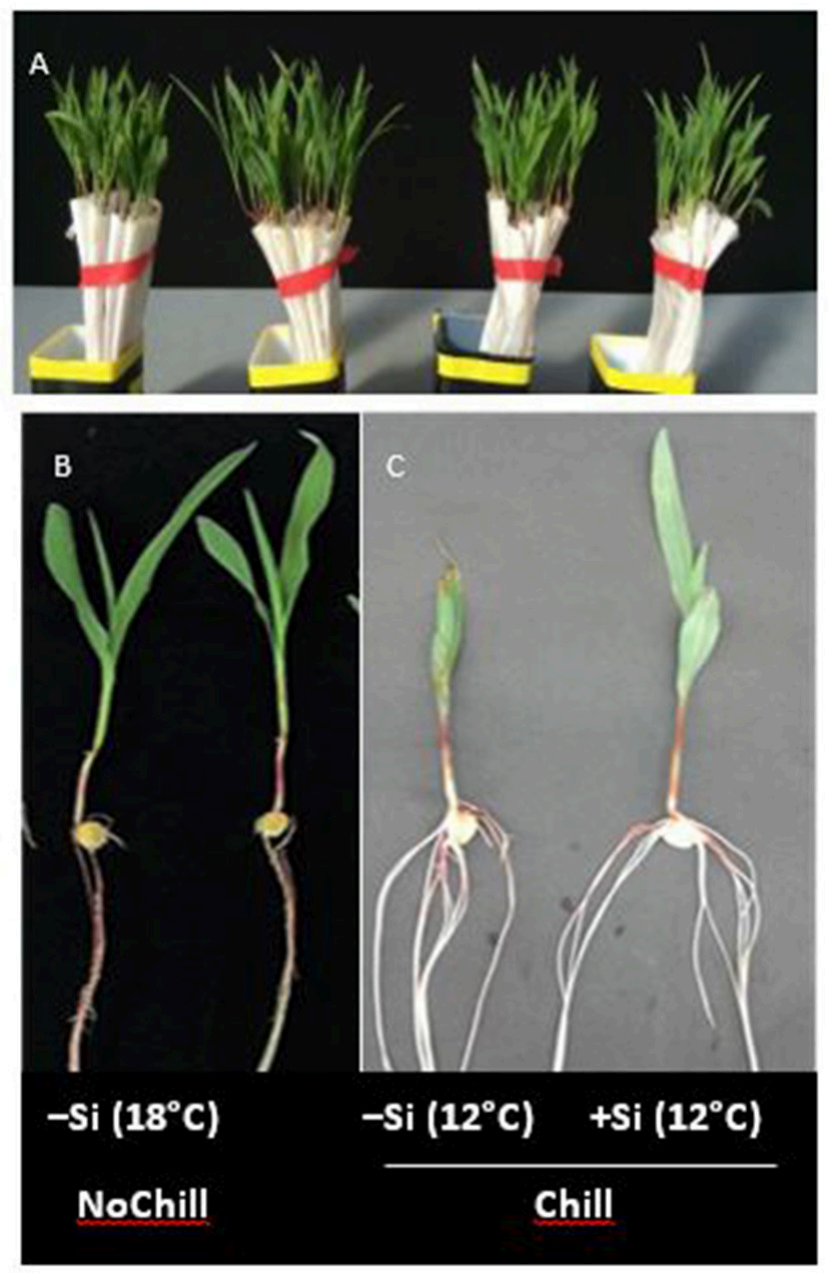
**(A–C)** Maize seedlings germinated in filter rolls for 7 d at 18°C (NoChill) or at 12°C (Chill) without (−Si) or with (+Si) after 3 d seed soaking in 0.1 mM Si (H_4_SiO_4_).

During the first 3 days of cultivation at 18°C, no significant treatment differences were recorded for germination rates (Figure 4). However, the subsequent 7 d–cold stress periods at 12°C induced a retardation in seedling growth, associated with a reduction in shoot and root biomass production by 53 and 60%, respectively, and intense necrotic and chlorotic leaf damage (Table 2) similar to the symptoms observed in soil culture (Figures 2A and 5). Silicon treatments reduced the necrotic leaf area by more than 90%. This was associated with increased levels of total antioxidants (+27%) and soluble sugars (+117%) in the leaf tissue. The nutritional status of Zn and Mn was significantly increased by the Si treatment and a trend for increased root and shoot biomass production was detectable after the cold period of 7 d (Table 2).

**Table 2:** Effect of Si seed soaking on biomass production, oxidative leaf damage [% necrotic leaf area], tissue concentrations of Zn, Mn, Si, soluble sugars, and total antioxidants in maize seedlings (cv Colisee) exposed to 7 days chilling stress at 12°C in a soil-free filter roll culture system.

	**–Si (18°C)**	**–Si (12°C)**	**+Si (12°C)**
Dry weight (Shoot) [g]	0.98 ± 0.11a	0.46 ± 0.06b	0.63 ± 0.27ab
Dry weight (Root) [g]	0.85 ± 0.10a	0.34 ± 0.04b	0.41 ± 0.05b
Zn (Shoot) [μg g^−1^ DW]	16.25 ± 1.63a	11.42 ± 1.15*c*	13.73 ± 1.38b
Zn (Root) [μg g^−1^ DW]	11.85 ± 1.20a	9.08 ± 0.92b	10.80 ± 1.09a
Mn (Shoot) [μg g^−1^DW]	3.23 ± 0.33a	1.56 ± 0.17b	2.63 ± 0.27a
Mn (Root) [μg g^−1^ DW]	2.12 ± 0.22a	0.99 ± 0.11b	1.43 ± 0.69ab
Total Antioxidants (Shoot) [%]	62.25 ± 6.24b	67.30 ± 6.74b	85.21 ± 8.53a
Necrotic leaf area [%]	0.50 ± 0.06*c*	25.25 ± 2.54a	2.35 ± 0.25b
Soluble Sugars (Shoot) [mg g ^−1^ FW]	28.15 ± 2.83a	12.12 ± 1.22b	26.23 ± 2.63a
Si (Shoot) [mg g ^−1^ DW]	0.86 ± 0.20b	0.63 ± 0.07*c*	1.32 ± 0.14a

*Means of five replicates. Significant differences (P < 0.05) are indicated by different characters*.

During the 7-days cold stress period, shoot and root concentrations of all important hormonal growth regulators (indole acetic acid—IAA, zeatin, gibberellic acid—GA), as well as stress–related hormones, such as abscisic (ABA), jasmonic and salicylic acids, declined significantly. However, after Si seed soaking, hormonal concentrations were restored comparable to those of unstressed plants or even further increased (shoot concentrations of IAA, gibberellic acid, and ABA, Table 3).

**Table 3:** Endogenous concentrations of phytohormones in maize seedlings (cv Colisee) grown for 7 days at 18 or 12°C, in a soil-free filter roll culture system with (+Si) or without (–Si) silicic acid seed-soaking (–Si) during a 3-day pre-germination period at 18°C.

**Tissue**	**Shoot**			**Root**		
**Temperature**	**18°C**	**12°C**	**12°C**	**18°C**	**12°C**	**12°C**
**Treatment**	**−Si**	**−Si**	**+Si**	**−Si**	**−Si**	**+Si**
**Phytohormones**	**[ng g**^**−1**^ **fresh weight]**					
IAA	76.77 ± 9.90a	28.14 ± 4.65b	102.12 ± 16.54a	31.45 ± 8.81a	12.76 ± 0.37b	40.10 ± 13.43a
Zeatin	3.24 ± 0.84a	1.10 ± 0.22b	3.62 ± 0.65a	1.29 ± 0.49a	0.40 ± 0.04b	1.21 ± 0.31a
ABA	50.63 ± 4.13b	31.02 ± 4.04*c*	109.56 ± 13.69a	46.56 ± 8.65a	14.97 ± 0.91b	48.78 ± 14.19a
GA	55.60 ± 10.29b	18.26 ± 3.14*c*	67.20 ± 9.77a	25.07 ± 6.27a	9.51 ± 0.70b	30.12 ± 10.49a
JA	0.44 ± 0.07a	0.28 ± 0.03b	0.42 ± 0.09a	ND	ND	ND
SA	44.89 ± 6.79a	16.78 ± 3.17b	56.27 ± 15.26a	21.96 ± 4.71a	9.53 ± 0.79b	28.94 ± 12.00a

*IAA, indole acetic acid; ABA, abcsisic acid; GA, gibberellic acid; JA, jasmonic acid; SA, salicylic acid. Means of three replicates. Significant differences (P < 0.05) are indicated by different characters. ND, Not Detectable*.

**Figure 6:**
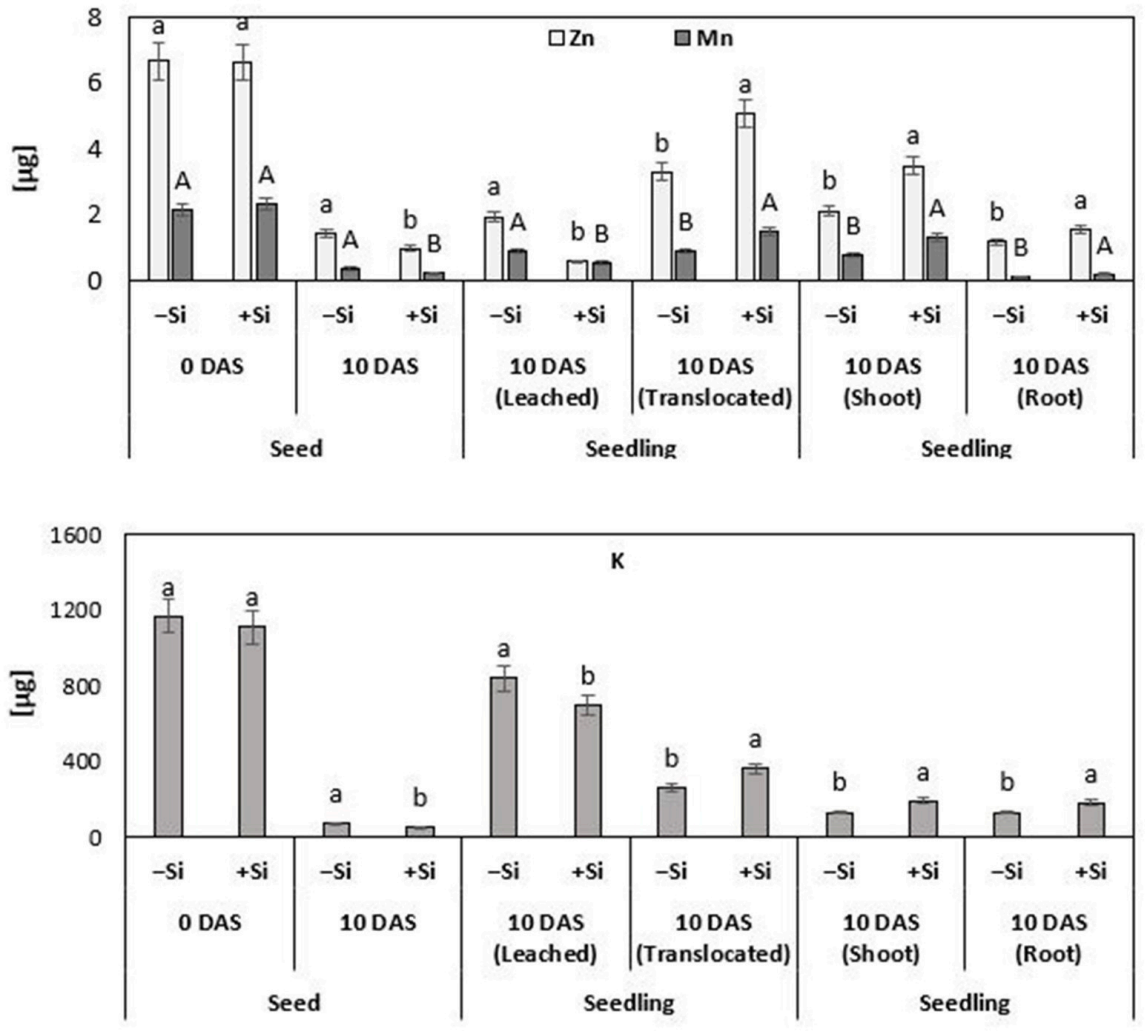
Fate and distribution of Zn, Mn, and K [μg seed^−1^ or μg seedling^−1^] during germination and seedling growth of maize (cv Colisee) exposed to chilling stress (12°C; 3–10 DAS) in a soil-free filter roll culture system with (+Si) or without (–Si) silicic acid seed soaking (–Si) during a 3-day pre-germination period at 18°C. Means of five replicates. Significant differences (*P* < 0.05) are indicated by different characters.

Since plant cultivation in this experiment was conducted in a nutrient–free culture system, lacking any additional Zn or Mn (Si treatment solution pre-purified by cation exchange chromatography) (Maksimovic et al., 2007; Pavlovic et al., 2013), it was possible to monitor the redistribution of seed–stored Zn and Mn during seedling development. Thus, any additional nutrient uptake via the roots was excluded. During the 10 days culture period, 50% of the seed Zn contents and 42% of seed Mn were translocated to the developing control seedlings. Interestingly, the translocation of micronutrient seed reserves was stimulated by the Si treatments, resulting in significantly higher total Zn (+67%) and Mn (+62%) contents at the end of the cold stress period, as compared with the untreated controls (Figure 6). By comparison with the remaining nutrient contents in the seeds, it was also possible to calculate the nutrient leaching losses. In the untreated control plants exposed to chilling stress, leaching accounted for 29% of the original seed Zn contents and for 41% of Mn. However, silicon treatment significantly reduced the large leaching losses of Zn and Mn by 70 and 48%, respectively (Figure 6).

Apart from the micronutrients, also the re-distribution of K was investigated, because of its well-established role as an indicator for nutrient leaching in response to impairment of tissue and membrane integrity (Cakmak and Marschner, 1987) and its function in cold stress protection (Wang et al., 2013). At the end of the cold stress period, untreated control seedlings had lost 72% of their K seed reserves by leaching and only 22% had been translocated to the developing seedling. Silicon treatments reduced these large leaching losses by 17% and increased the K contents of the seedling by 38% (Figure 6). For other tested macronutrients, such as P, Mg, and Ca, no significant treatment effects were recorded (data not shown). However, Si not only reduced the leaching losses and improved the translocation of seed reserves of mineral nutrients to growing tissues. Additional selective effects were detectable for the mineral nutrient ratio between shoots and roots. While Si pre-treatments increased the Mn and K contents in shoots and roots to a similar extent (Mn: +68–77%; K: +38–46%), Si preferentially increased the Zn shoot contents by 64%, but less, only by 34%, in the root tissue (Figure 6).

### Field experiment

To investigate the benefit of Zn/Mn and Si starter treatments on maize performance under on-farm conditions, a field experiment was established. Early sowing on April 22nd resulted in cold stress during germination and early growth. Due to low temperatures (13.6°C) and high precipitation (469 mm) during April and May, emergence and seedling growth were heavily affected by cold stress, but also by oxygen limitation and *Aspergillus niger* infections on the cold and wet silty-loam soil. By the mid of May, at 41 DAS (BBCH 15, stage 1), an emergence rate of only 44% was recorded in the untreated control variant (Table 1). However, emergence was significantly increased by the Zn/Mn seed dressing (+12%) and particularly by seed soaking with KSi (+28%). To account for a potential water priming effect of the seed soaking treatment (Lutts et al., 2016), also a water-soaked control was included, which increased emergence by 8% (Table 4).

**Table 4:** Emergence, Zn/Mn status (DAS), and final biomass yield of field-grown silo maize (Rolandinio) on a silty loam soil pH 6.9 at the experimental station “Ihinger Hof” University of Hohenheim with underfoot placement of di-ammonium phosphate (29 kg N, 32 kg P ha^−1^) and stabilized ammonium sulfate fertilization (161 kg N ha^−1^) with or without Zn/Mn seed dressing (Lebosol Mn500 SC, Zn700 SC), potassium silicate [1 mM] seed priming, water priming of foliar Si application of Si as (16 L Si [1 mM] + 100 ml Greemax® ha^−1^).

**Seed treatment**	**Application ode**	**Emergence 41 DAS [%]**	**Yield [t DM ha^−1^]**	**Zn status (45DAS) [μg g DW^−1^]**	**Mn status (45 DAS) [μg g DW^−1^]**
Untreated	–	44.00 ± 6.00d	7.14 ± 0.46d (16.10 ± 1.0b)	32.00 ± 2.60b	48.00 ± 4.00a
Zn/Mn	Seed–dressing	56.00 ± 7.00b	11.07 ± 0.79b (16.40 ± 1.2b)	65.00 ± 5.40a	56.00 ± 4.50a
Water	Priming	52.00 ± 9.00c	8.10 ± 0.81c (17.2 ± 1.60ab)	49.00 ± 4.08a	57.00 ± 4.75a
K_2_SiO_4_	Priming	72.00 ± 15.00a	12.89 ± 0.74a (17.8 ± 1.0a)	59.00 ± 4.90a	61.00 ± 5.08a
untreated	Si foliar	46.00 ± 7.00d	7.60 ± 0.37d (16.6 ± 3.40ab)	36.00 ± 3.00b	51.00 ± 4.25a

*Yield determinations with (in brackets) and without re-sowing by the end of May 2016. Means of five replicates per treatment. Significant differences (P < 0.05) are indicated by different characters*.

At 49 DAS (BBCH 17, stage 1), a mineral nutrient analysis was conducted for the youngest fully developed leaves. In the control variant, the Zn–nutritional status was low (Table 4) as expected, and K and P were even below the deficiency thresholds. Mg, Cu, and Mn supply, by contrast, was sufficient (Supplementary Table 2). Similar to the pot experiment, both, Zn/Mn and Si treatments significantly increased the Zn concentrations to a sufficient level of 65 and 59 μg g^−1^ dry matter (Bergmann, 1988). A trend for an improved status was recorded also for the remaining nutrients (Supplementary Table 2). The protective effects of Zn/Mn and Si starter treatments were finally reflected in substantially higher biomass yield of the heavily cold-affected plots by 56 and 82%, respectively, when calculated according to the plant density determined at 41 DAS. In the Si treatments, 25% of the yield increase could be attributed to a water priming effect. Foliar Si application had no significant impact on final biomass yield (Table 4). However, due to the extremely low emergence (44%), particularly in the untreated plots, re-sowing was performed in the most heavily affected parts, by the mid of May after the end of the stress period, to provide comparable inter-plant competition for light, water, and nutrients within the rows during the rest of the culture period. But even including the non-stressed plants after re-sowing into the yield calculation, a significant yield increase (+10.6%) was measured only for the Si seed priming variant (Table 4).

## Discussion

### Si mimics cold stress-protective effects of Zn/Mn starter applications

Similar to earlier reports (Imran et al., 2013; Bradáčová et al., 2016), starter treatments by seed dressing or seed priming with Zn and Mn significantly increased cold tolerance of maize in the pot experiment with controlled root-zone temperature (Figure 2). The 14-d chilling treatments with 12°C root zone temperature induced Zn and Mn deficiencies in the soil-grown maize seedlings (Figure 3). This seems to be not primarily related to Zn/Mn availability in the soil, since Imran et al. (2013) demonstrated that Zn and Mn accumulation in the shoot tissue was suppressed during the cold stress period, even with freely available nutrient supply in a hydroponic culture system. Only Zn and Mn uptake before the onset of the stress period via seed priming, seed dressing or fertigation, could increase shoot micronutrient accumulation above the deficiency thresholds, detectable even after the end of the 2-weeks cold stress period (Figure 3). This is in line with findings of Engels and Marschner (1992, 1996), demonstrating that limited Zn and Mn shoot accumulation in maize exposed to low root zone temperatures was particularly dependent on cold-stress effects affecting nutrient uptake and root activity.

In accordance with the low Zn/Mn status, the cold-stressed control plants exhibited various symptoms characteristic for Zn and Mn limitation (Cakmak, 2000), such as chlorosis and oxidative leaf damage, stunted shoot, and root growth (Figure 2) reduced activity of enzymes involved in ROS detoxification with micronutrients as co-factors (SODs, PODs), and impaired biosynthesis of phenolic antioxidants (Table 1) which depends on Cu and Mn cofactors (Datnoff et al., 2007). Consequently, excessive accumulation of ROS (Table 1) resulted in oxidative damage of plant tissues (Figure 2) considered as one of the major constraints for cold-stressed plants (Baek and Skinner, 2012; Saeidnejad et al., 2012). Supplementation of Zn and Mn via seed priming (Imran et al., 2013) or seed dressing were able to overcome Zn/Mn deficiency (Figure 3) and largely mitigated the related stress symptoms described above. Surprisingly, also starter fertigation or seed soaking with Si completely mimicked all cold-stress-protective effects of Zn/Mn starter applications (Figures 2, Table 1). Silicon increased the Zn/Mn status, activities of ROS detoxification enzymes, and accumulation of antioxidants to a comparable level as the Zn/Mn treatments (Figures 3, Table 1) although Si was applied as free silicic acid (H_4_SiO_4_), pre-purified via cation exchange chromatography, and no Zn or Mn was detectable in the application solution.

Also in the field experiment, seed treatments with Si and Zn Mn improved emergence and the micronutrient status (particularly Zn) of maize seedlings, exposed to sub-optimal germination temperatures and cold and wet soil conditions by early sowing at the mid of April (Table 4). A certain protective effect was recorded also for the control treatment with water-primed seeds but less expressed as compared with Si seed priming. This is in line with the well-documented beneficial effects of water priming on seed germination by metabolic pre-activation (Lutts et al., 2016). In accordance with earlier observations (Imran et al., 2013; Bradáčová et al., 2016), application of cold stress protectants was ineffective after the onset of the stress period, i.e., when Si was supplied by the foliar application (Table 4). By contrast, silicon seed priming was the most effective treatment and increased emergence by 64% as compared with the untreated control, which translated into a significant increase in final yield in both scenarios of yield determination (with and without re-sowing after the end of the cold stress period; Table 4). Similar effects have been reported also in two additional field experiments conducted under comparable climatic conditions when the micronutrient status of the maize seedlings was increased by direct supplementation of Zn, Mn, and Fe via seed priming. In these field trials, marketable grain yields increased by 13–15% (Imran et al., 2013). The surprisingly long-lasting effects of the starter treatments with cold stress protectants may be related to the intense stimulation of root growth (Figure 2D) which has an advantage for plant performance not only during the stress period but also during the recovery phase and under more favorable growth conditions.

### Silicon reduces leaching losses and promotes utilization of Zn/Mn seed reserves

The striking similarity of Zn/Mn and Si effects on cold-stressed maize seedlings raises the question whether Si exerts its protective effects via improvement of the plant micronutrient (Zn/Mn) status. Restoration of cold stress-induced root growth inhibition was among the most apparent effects of Si or Zn/Mn applications (Figure 2E). Of course, root growth stimulation can contribute to improved nutrient acquisition in general, as demonstrated for increased shoot accumulation of P, K, Mg, Ca, Fe, Zn, Mn, and Cu recorded after the cold stress period in Si-treated maize plants (Figure 3). However, Si was able to improve selectively the Zn/Mn status of maize seedlings, already during the first week of the cold stress period, before a marked stimulation of root growth was detectable (Table 2). Moreover, this effect was observed in a soil–free culture system excluding the option for further Zn/Mn root uptake from the external medium (Figure 5). This implicates that the improved micronutrient status of the Si-treated maize seedlings exposed to low-temperature stress cannot be exclusively attributed to Si-induced root growth stimulation but involves also Si effects counteracting nutrient leaching and promoting internal distribution of Zn and Mn. Imran et al. (2015) demonstrated that seed reserves can cover the Zn and Mn demand of maize seedlings for about 2–3 weeks. However, cold stress is a well-documented stress factor leading to electrolyte leakage via oxidative membrane damage (Bewley and Black, 1994), which can limit the seed reserves of mineral nutrients. Accordingly, our study revealed large leaching losses of 30–40% for the Zn/Mn seed reserves and even 70% for K, as another mineral nutrient with cold-protective functions (Wang et al., 2013), during the first 10 days of seedling development after a 12°C cold stress period of 7 days (Figure 6). Seed soaking with Si dramatically reduced the leaching losses by 70% (Zn), 50% (Mn), and 15% (K), leading to an improved nutrient supply to the developing seedling. This effect may be attributed to the well-documented protective functions of Si against oxidative membrane damage (He et al., 2010). However, Si seed soaking increased the root and shoot contents of Mn by 77 and 68%, respectively, while the root contents of Zn were increased only by 34%, but by 64% in the shoot tissue (Figure 6). This indicates a selective effect of Si on the root/shoot distribution of Zn. A similar improved Zn status by Si treatment has been reported for Zn-deficient soybean plants (Pascual et al., 2016).

Different mechanisms have been proposed for effects of Si, improving internal Zn availability in Zn-deficient plants. Increased production of phenolics with metal chelating properties induced by Si treatments (Pavlovic et al., 2013), as observed also in the present study (Table 1) may increase internal mobility and transport of Zn within the plant. This may be related to the improved Mn status of Si treated plants (Figure 3) as an important enzymatic cofactor for the biosynthesis of phenolics (Datnoff et al., 2007). In later stages of plant development, the same mechanism may be responsible also for the remobilization of Zn sequestered in the apoplast together with iron plaques (Chen et al., 1980), as similarly demonstrated for apoplastic Fe remobilization in cucumber (Pavlovic et al., 2013). Bityutskii et al. (2014) could not confirm this interaction, but in that study, plants were grown in nutrient solution without Zn supply, which may have prevented the accumulation of apoplastic Zn pools. Other studies suggest direct Si-metal interactions counteracting apoplastic metal immobilization and supporting metal transport in plants (Pavlovic et al., 2013; Hernandez–Apaolaza, 2014; Stevic et al., 2016). Effects of Si on the expression of metal acquisition and transport genes have been reported by Pavlovic et al. (2013, 2016), but the underlying mechanisms are unknown.

### Si restores the levels of hormonal growth regulators in cold-stress-affected maize seedlings

Due to the impairment of Zn/Mn-dependent ROS detoxification systems, induced by limited Zn/Mn availability in cold-stressed plants (Figures 2, 3) excessive ROS accumulation causes oxidative damage, leading to leaf chlorosis and necrosis (Figure 2). This is associated with an impairment of photosynthesis, resulting in a reduced root allocation of assimilates required for root development, as previously reported also by Sowinski et al. (1998). Moreover, excessive production of ROS can promote oxidative degradation of indole acetic acid and result in a 50% reduction of IAA contents in Zn-deficient *Phaseolus vulgaris*, which could be restored by Zn fertilization. Therefore, auxin deficiency was considered as another important factor for growth limitation in Zn deficient plants (Cakmak et al., 1989). Similarly, in our study cold stress induced, both, Zn/Mn limitation (Table 1) and a 60% reduction of IAA accumulation in the shoot and root tissue (Table 3) associated with inhibition of shoot and root growth and all symptoms were reverted by exogenous Si application (Figure 2). More recent studies suggest that cold stress additionally affects root growth via inhibition of PIN2 and PIN3-mediated basipetal auxin transport within the roots (Shibasaki et al., 2009).

Shoot growth in cold-stressed plants seems to be also affected by a reduction of bioactive growth-promoting gibberellic acid (GA) levels, leading to an increased abundance of nuclear DELLA-protein growth repressors via a signaling pathway involving CBF/DREB1 transcription factors (Miura and Furumoto, 2013; Eremina et al., 2016). Accordingly, in our study GA levels in shoot and roots of cold-stressed maize seedlings declined by ~60–70%. This effect was completely reverted by Si seed soaking (Table 3). In line with this observation, in *Arabidopsis thaliana* it was demonstrated that cold stress stimulates GA degradation by upregulation of the GA 2-oxidase gene and simultaneously impairs GA-biosynthesis by repressing the GA 20-oxidase gene (Eremina et al., 2016). For Zn-deficient plants, reduced GA concentrations have been recorded (Suge et al., 1986; Sekimoto et al., 1997) similar to the reduction in IAA levels reported by Cakmak et al. (1989). More recently, it was shown that various steps of GA biosynthesis depend on the presence of IAA (Ross et al., 2001). Therefore, the observed reduction of GA levels in cold-stressed maize seedlings (may be a consequence of auxin deficiency, resulting from the limited Zn supply caused by low temperature stress. Interestingly, it was shown that exogenous application of IAA and GA could increase the accumulation of flavonoids and other phenolics in buckwheat (*Fagopyrum esculentum*) seedlings (Park et al., 2017). This observation is in line with the increased production of phenolics and antioxidants, induced by Si application in cold-stressed maize seedlings (Table 1) that may be triggered by the increased IAA and GA levels in the respective plants (Table 3).

Similar to auxin and GA, also the levels of the metabolically active cytokinin form “zeatin,” with functions in stimulation of cell division and cell expansion, declined in cold-stressed maize seedlings (Table 3). A similar decline had been previously reported for *Arabidopsis*, rice and, wheat (Eremina et al., 2016). In rice, this was associated with a significant downregulation of gene expression related to cytokinin biosynthesis (Maruyama et al., 2014). Accordingly, external application of cytokinins increased cold tolerance in *Arabidopsis* (Jeon et al., 2010; Shi et al., 2012). In our study, cold-stress protection by Si application was also associated with increased zeatin concentrations in the root and shoot tissue (Table 3).

Taken together, the results demonstrated that cold stress significantly reduced the internal concentrations of the most important hormonal regulators of plant growth. Furthermore, the cold-protective effect of Si application was related to a restoration of hormonal levels comparable to those of unstressed plants (Table 3).

### Si increases the levels of hormonal stress regulators in cold stress-affected maize seedlings

The Si treatments influenced also the levels of abscisic (ABA) salicylic (SA) and jasmonic acids (JA), known as hormones more directly involved in the regulation of abiotic and biotic stress responses (Table 3). These hormones have been also implicated in cold stress signaling (Miura and Furumoto, 2013; Eremina et al., 2016). Improved cold acclimation and increased cold tolerance by exogenous applications of ABA, SA, and JA have been reported for various plant species (Horváth et al., 2007; Kumar et al., 2008; Eremina et al., 2016; Hu et al., 2017). Mutants affected in ABA, SA, and JA metabolism show altered responsiveness to cold stress (Eremina et al., 2016). Accordingly, in our study, exposure of maize, as cold-sensitive plant species to a 7-days cold period, significantly decreased the levels of ABA, SA, and JA. The cold-protective effect of Si starter treatments was associated with an increase of ABA and to a smaller extent also of SA and JA, particularly in the shoot (Table 3). Abscisic acid is considered as a central regulator of cold stress responses in plants and seems to regulate the adaptive expression of cold-related genes with cold-protective functions via CBF-dependent and independent pathways, in cross-talks involving also SA and JA (Szalai et al., 2011; Eremina et al., 2016). Direct links between ABA and induction of oxidative stress defense enzymes, such as SOD in cold-stressed plants similar to our study (Figure 3) have been reported by Kumar et al. (2008), Szalai et al. (2011), and Li and Zhang (2012). Moreover, the cold stress-induced accumulation of cryo-protectants, such as proline (Figure 3) has been linked with a reduction of leaching losses via ABA-induced protection against oxidative membrane damage (Chen and Li, 2002). Proline biosynthesis at least partially depends on ABA signaling (Szabados and Savouré, 2010). Increased ABA levels in the Si-treated plants may be also related to the improved Zn-nutritional status induced by the Si starter treatments (Figure 3). Accordingly, Cakmak et al. (1989) and more recently Wang et al. (2012) reported that Zn deficiency reduced the ABA levels in *Phaseolus vulgaris* and in apple rootstocks.

**Figure 7:**
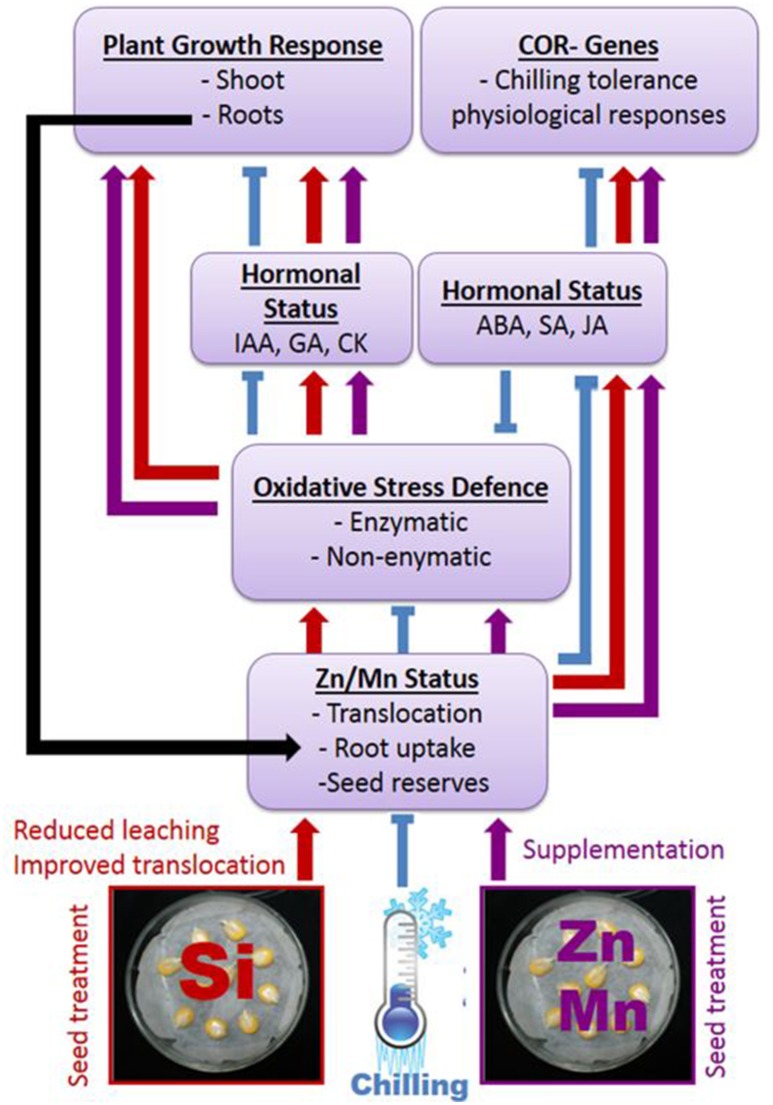
Proposed interactions between Si or Zn/Mn seed treatments and expression of chilling tolerance in maize. Arrows indicate stimulation, t–shaped lines inhibition of the respective processes.

The surprisingly distinct effects of the Si treatments on the hormonal balances (Table 3) and the timing of hormonal changes just at the beginning of detectable growth responses (Table 2) are clear indicators for the proposed interactions of the Si amendments with hormonal cold stress signaling. Nevertheless, the obvious interactions of Si with hormonal balances require further investigations considering quantitative changes during plant development and a higher spatial resolution, since it is known that local changes in hormone concentrations at the cellular level control adaptive growth and development (Eremina et al., 2016). A conceptual model of the proposed interactions between Si, micronutrients, and hormones mediating chilling tolerance in maize seedlings and its relation to plant growth is presented in Figure 7 and summarizes our findings.

## Concluding remarks

The findings of the present study suggest that induced deficiency of Zn and Mn as a consequence of leaching during early development and limited root growth and activity is a major factor determining the sensitivity of young maize plants to chilling stress, with options for mitigation by supplementing germinating seeds with Zn, Mn, or Si. In this context, the protective effect of Si treatments is related to an improved Zn and Mn status, starting already during germination with a protective effect of Si against leaching losses of micronutrient seed reserves and improved translocation to the developing seedling. The improved micronutrient status can at least partially explain the ability of the plants to maintain a balanced hormonal (IAA, GA, cytokinin) status that restores plant growth and particularly root development, which allows further nutrient uptake. The improved micronutrient status also helps to increase the expression of enzymatic (SOD, POD) and non-enzymatic (phenolic antioxidants) defense systems against cold-induced oxidative stress. Finally, it stimulates the accumulation of stress hormones (ABA, SA, JA), potentially priming various physiological adaptations to chilling stress via expression of cold response genes. It remains to be established whether this applies also to protective effects of Si against other abiotic stresses, such as drought or salinity, which are also associated with oxidative stress. Remarkably, the improved micronutrient status by Si treatment under low temperature stress is not only restricted to controlled lab conditions. The preliminary field experiment demonstrated that the observed protective effects of Si starter treatments on seedling performance can translate into improved yields in agricultural practice and requires further investigation considering different soil types and maize varieties.

## Author contributions

NM, GN, and MW: conceived and designed the experiments; NM: conducted the experiments, collected the data, and wrote the manuscript with GN and UL; FW, BH, and NM: developed and performed the UHPLC-MS analysis of phytohormones. All authors approved the final manuscript.

### Conflict of interest statement

The authors declare that the research was conducted in the absence of any commercial or financial relationships that could be construed as a potential conflict of interest.

## Abbreviations

Zn: zinc
Si: silicon
Mn: manganese
IAA: indole acetic acid
SA: salicylic acid
JA: jasmonic acid
GA: gibberellin
COR: cold response
RZT: root zone temperature
DAS: days after sowing
ROS: reactive oxygen species
SOD: superoxide dismutase
POD: peroxidase.
