# Supplementary material for: Silicon Improves Chilling Tolerance During Early Growth of Maize by Effects on Micronutrient Homeostasis and Hormonal Balances
Source: Front Plant Sci. 2018 Apr 26;9:420. doi: 10.3389/fpls.2018.00420 (PMC5935009; doi:10.3389/fpls.2018.00420)
Supplement: Supplementary file 1 [file Data_Sheet_1.docx]

**Supplementary DATA set**

**Table 1.** Chemical and physical properties of the soil material from IHO

| pH-Value (CaCl_2_-solution) | 6.5 |
| --- | --- |
| Humus | 2.12 % |
| C_org_ | 1.23 % |
| Carbonate-C | < 0.20 % |
| C_total_ | 1.27 % |
| N | 0.18 % |
| P (CAL-extract VDLUFA) | 83 mg P kg^-1^ |
| K (CAL-extract VDLUFA) | 149 mg K kg^-1^ |
| Mg (CaCl_2_-extract VDLUFA) | 200 mg Mg kg^-1^ |
| Sand (63 -2000 µm) | 2 % |
| Silt (2 – 63 µm) | 71 % |
| Clay (< 2 µm) | 27 % |

**Table 2.** Mineral nutrient analysis at 49 days after sowing (BBCH (Code 17) stage 1), of the youngest fully developed leaves

| Treatment | Zn  (µg g^-1^ DM) | Mn  (µg g^-1^ DM) | Cu  ((µg g^-1^ DM) | K  (µg g^-1^ DM) | Mg  (µg g^-1^ DM) | P  (µg g^-1^ DM) |
| --- | --- | --- | --- | --- | --- | --- |
| No treatment | 32 B | 48 A | 8 A | 15.5 A | 2.6 A | 1.13 B |
| Zn/Mn seed dressing | 65 A | 56 A | 10 A | 16.0 A | 3.3 A | 1.22 A |
| KSi seed soaking | 59 A | 61 A | 11 A | 15.6 A | 2.7 A | 1.20 A |


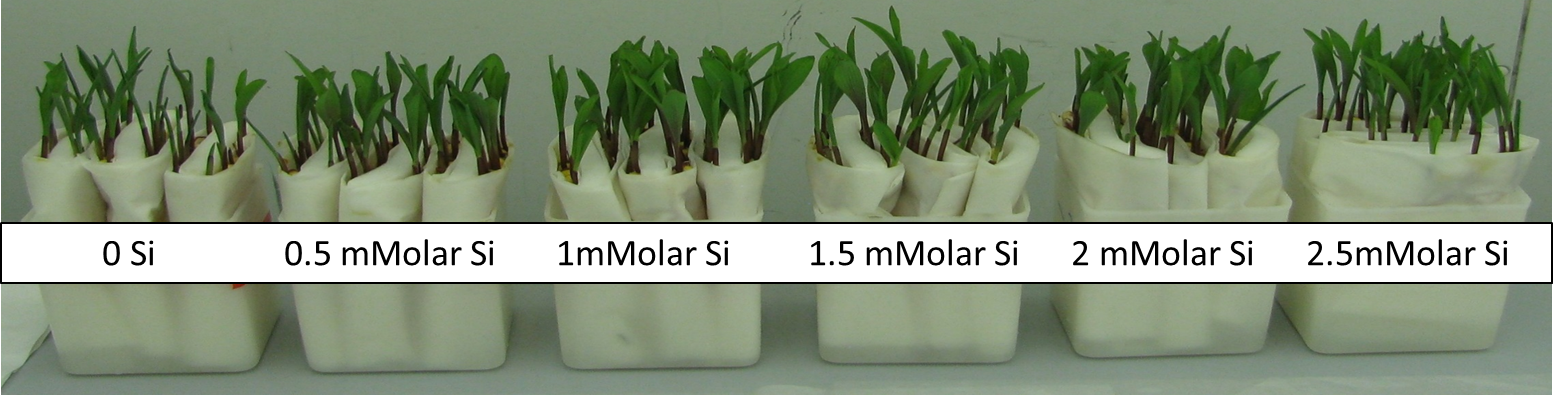


**Figure 1.** A pilot experiment was done to determine the optimum Si concentration for priming seed experiment.
